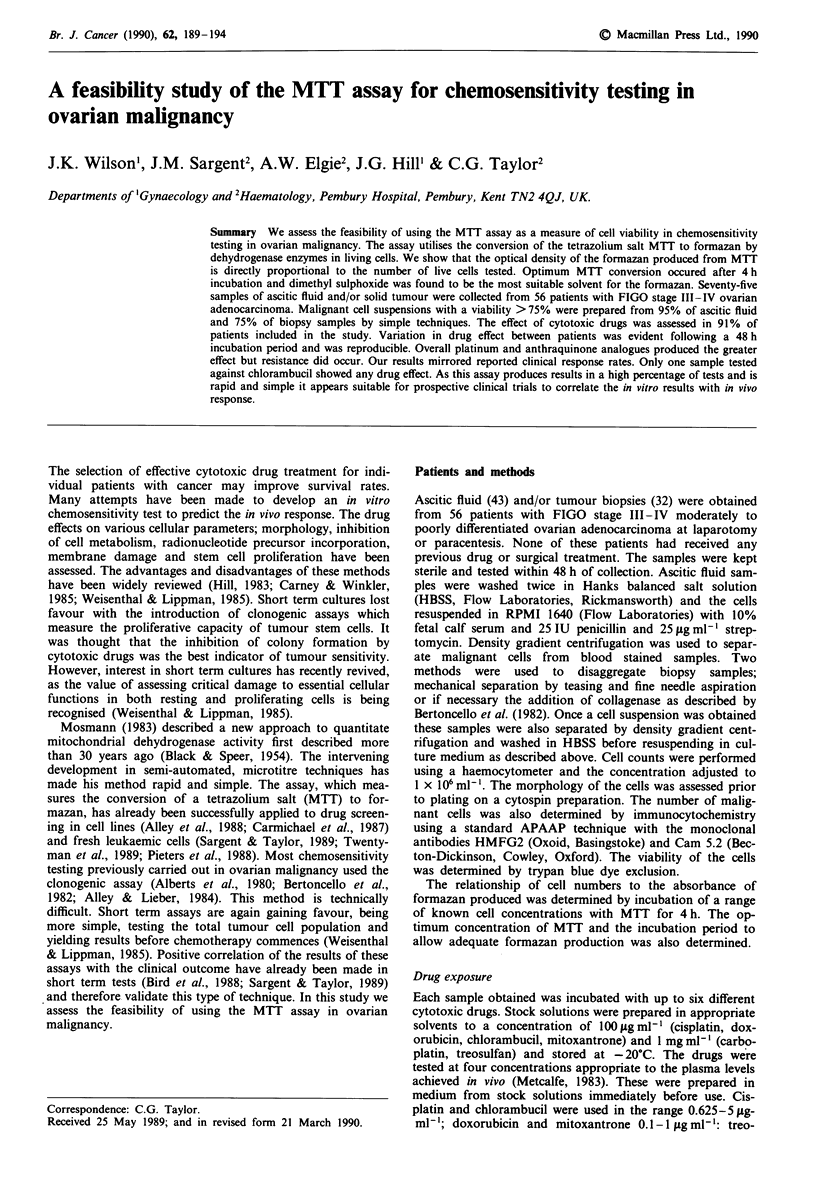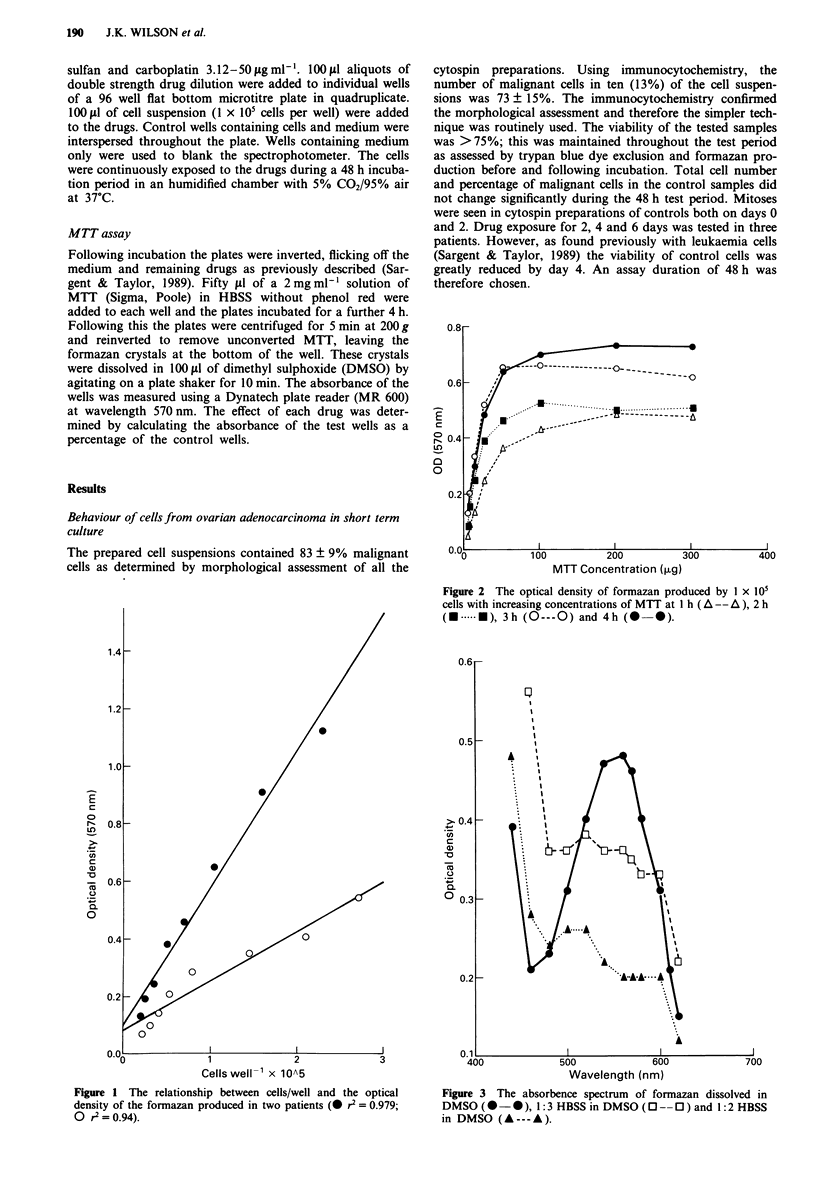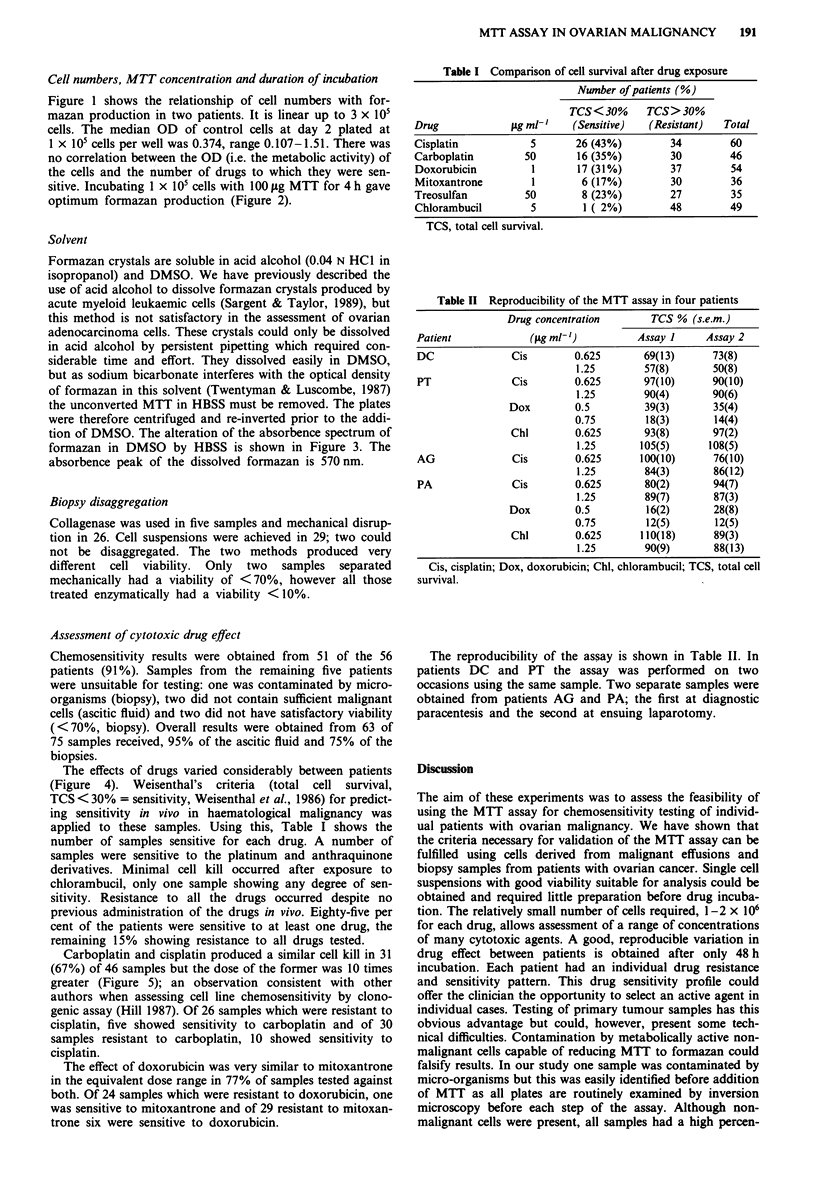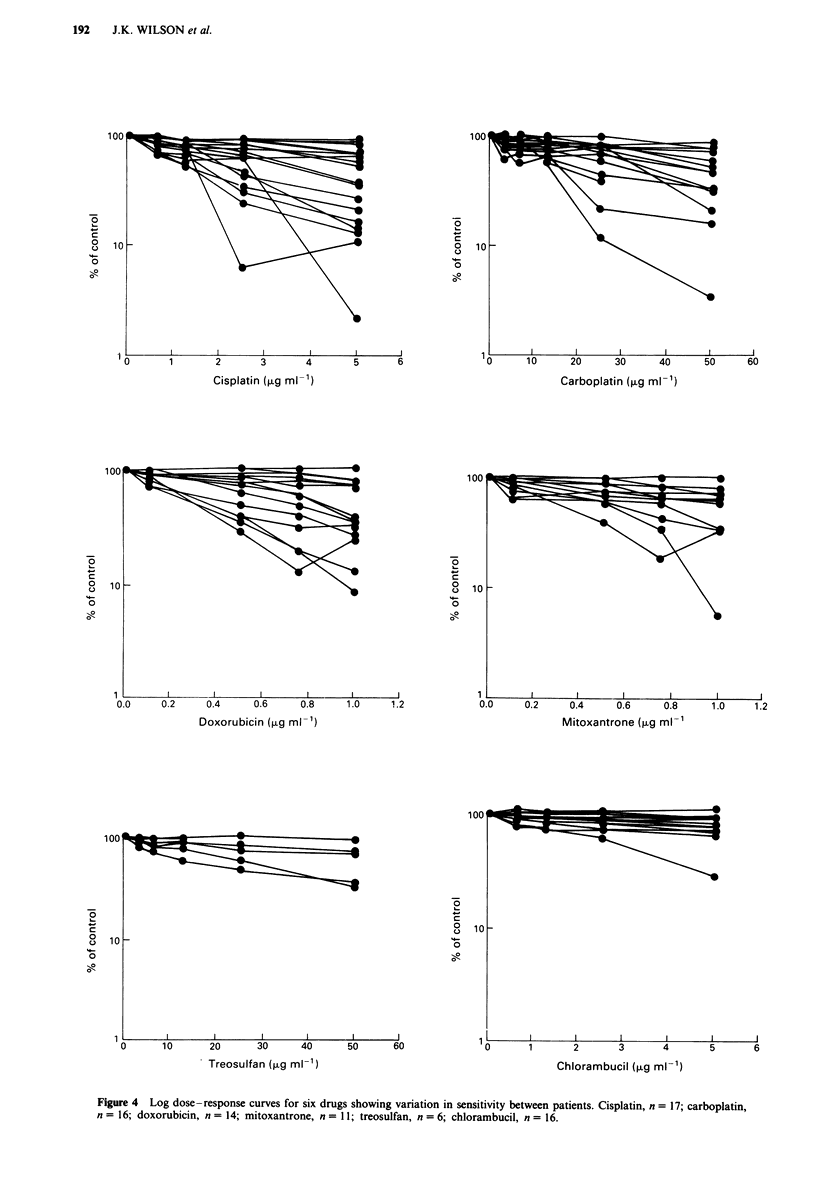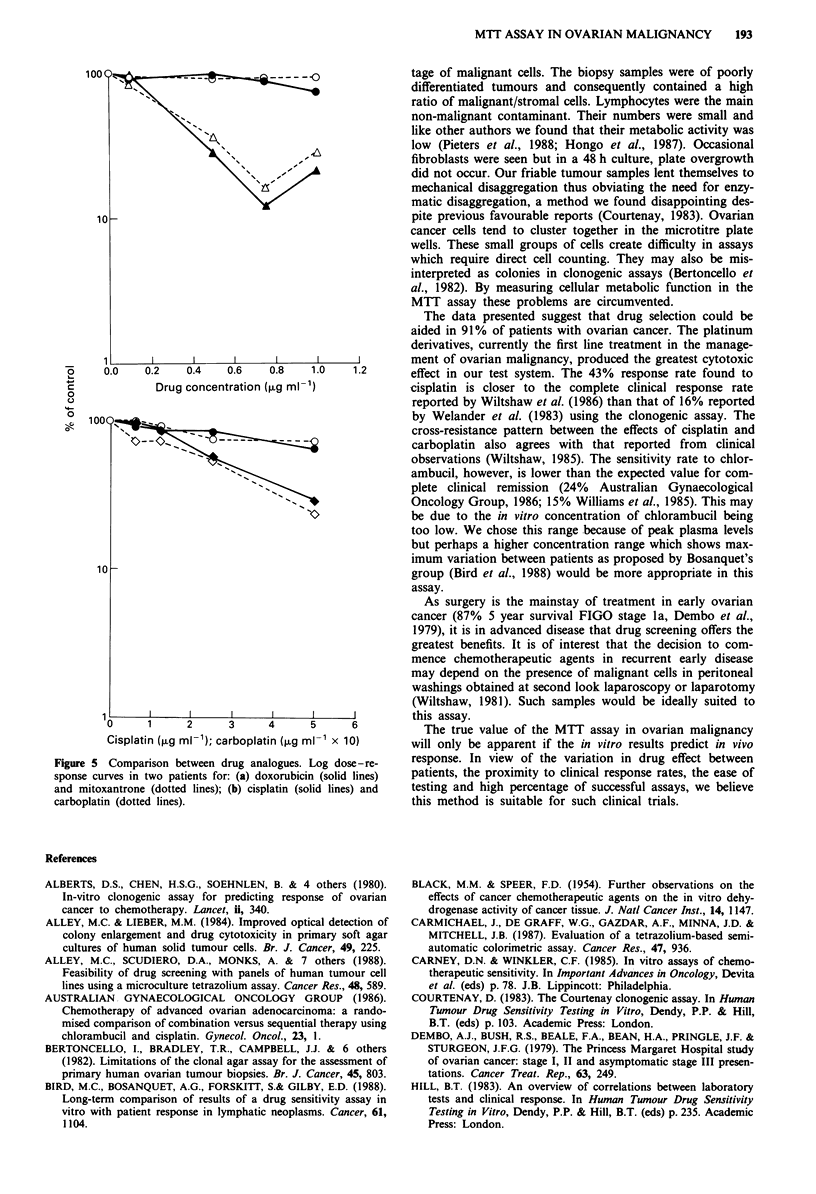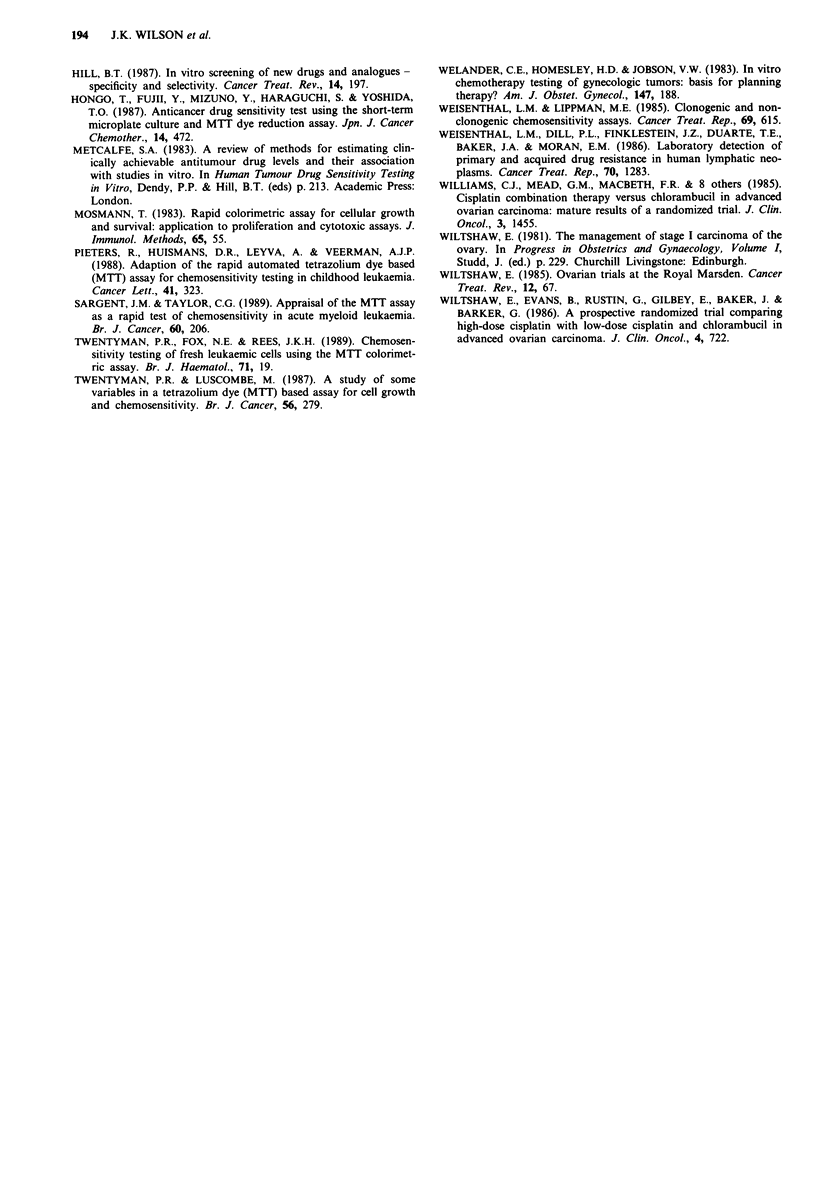# A feasibility study of the MTT assay for chemosensitivity testing in ovarian malignancy.

**DOI:** 10.1038/bjc.1990.258

**Published:** 1990-08

**Authors:** J. K. Wilson, J. M. Sargent, A. W. Elgie, J. G. Hill, C. G. Taylor

**Affiliations:** Department of Gynaecology, Pembury Hospital, Kent, UK.

## Abstract

We assess the feasibility of using the MTT assay as a measure of cell viability in chemosensitivity testing in ovarian malignancy. The assay utilises the conversion of the tetrazolium salt MTT to formazan by dehydrogenase enzymes in living cells. We show that the optical density of the formazan produced from MTT is directly proportional to the number of live cells tested. Optimum MTT conversion occurred after 4 h incubation and dimethyl sulphoxide was found to be the most suitable solvent for the formazan. Seventy-five samples of ascitic fluid and/or solid tumour were collected from 56 patients with FIGO stage III-IV ovarian adenocarcinoma. Malignant cell suspensions with a viability greater than 75% were prepared from 95% of ascitic fluid and 75% of biopsy samples by simple techniques. The effect of cytotoxic drugs was assessed in 91% of patients included in the study. Variation in drug effect between patients was evident following a 48 h incubation period and was reproducible. Overall platinum and anthraquinone analogues produced the greater effect but resistance did occur. Our results mirrored reported clinical response rates. Only one sample tested against chlorambucil showed any drug effect. As this assay produces results in a high percentage of tests and is rapid and simple it appears suitable for prospective clinical trials to correlate the in vitro results with in vivo response.


Br. J. Cancer (1990), 62, 189-194 © Macmillan Press Ltd., 1990

### A feasibility study of the MTT assay for chemosensitivity testing in ovarian malignancy

J.K. Wilson[1], J.M. Sargent[2], A.W. Elgie[2], J.G. Hill[1] & C.G. Taylor[2]

*Departments of [1]Gynaecology and [2]Haematology, Pembury Hospital, Pembury, Kent TN2 4QJ, UK.*

**Summary** We assess the feasibility of using the MTT assay as a measure of cell viability in chemosensitivity testing in ovarian malignancy. The assay utilises the conversion of the tetrazolium salt MTT to formazan by dehydrogenase enzymes in living cells. We show that the optical density of the formazan produced from MTT is directly proportional to the number of live cells tested. Optimum MTT conversion occured after 4 h incubation and dimethyl sulphoxide was found to be the most suitable solvent for the formazan. Seventy-five samples of ascitic fluid and/or solid tumour were collected from 56 patients with FIGO stage III-IV ovarian adenocarcinoma. Malignant cell suspensions with a viability >75% were prepared from 95% of ascitic fluid and 75% of biopsy samples by simple techniques. The effect of cytotoxic drugs was assessed in 91% of patients included in the study. Variation in drug effect between patients was evident following a 48 h incubation period and was reproducible. Overall platinum and anthraquinone analogues produced the greater effect but resistance did occur. Our results mirrored reported clinical response rates. Only one sample tested against chlorambucil showed any drug effect. As this assay produces results in a high percentage of tests and is rapid and simple it appears suitable for prospective clinical trials to correlate the *in vitro* results with *in vivo* response.

The selection of effective cytotoxic drug treatment for individual patients with cancer may improve survival rates. Many attempts have been made to develop an *in vitro* chemosensitivity test to predict the *in vivo* response. The drug effects on various cellular parameters; morphology, inhibition of cell metabolism, radionucleotide precursor incorporation, membrane damage and stem cell proliferation have been assessed. The advantages and disadvantages of these methods have been widely reviewed (Hill, 1983; Carney & Winkler, 1985; Weisenthal & Lippman, 1985). Short term cultures lost favour with the introduction of clonogenic assays which measure the proliferative capacity of tumour stem cells. It was thought that the inhibition of colony formation by cytotoxic drugs was the best indicator of tumour sensitivity. However, interest in short term cultures has recently revived, as the value of assessing critical damage to essential cellular functions in both resting and proliferating cells is being recognised (Weisenthal & Lippman, 1985).

Mosmann (1983) described a new approach to quantitate mitochondrial dehydrogenase activity first described more than 30 years ago (Black & Speer, 1954). The intervening development in semi-automated, microtitre techniques has made his method rapid and simple. The assay, which measures the conversion of a tetrazolium salt (MTT) to formazan, has already been successfully applied to drug screening in cell lines (Alley *et al.*, 1988; Carmichael *et al.*, 1987) and fresh leukaemic cells (Sargent & Taylor, 1989; Twentyman *et al.*, 1989; Pieters *et al.*, 1988). Most chemosensitivity testing previously carried out in ovarian malignancy used the clonogenic assay (Alberts *et al.*, 1980; Bertoncello *et al.*, 1982; Alley & Lieber, 1984). This method is technically difficult. Short term assays are again gaining favour, being more simple, testing the total tumour cell population and yielding results before chemotherapy commences (Weisenthal & Lippman, 1985). Positive correlation of the results of these assays with the clinical outcome have already been made in short term tests (Bird *et al.*, 1988; Sargent & Taylor, 1989) and therefore validate this type of technique. In this study we assess the feasibility of using the MTT assay in ovarian malignancy.

Correspondence: C.G. Taylor.
Received 25 May 1989; and in revised form 21 March 1990.

### Patients and methods

Ascitic fluid (43) and/or tumour biopsies (32) were obtained from 56 patients with FIGO stage III-IV moderately to poorly differentiated ovarian adenocarcinoma at laparotomy or paracentesis. None of these patients had received any previous drug or surgical treatment. The samples were kept sterile and tested within 48 h of collection. Ascitic fluid samples were washed twice in Hanks balanced salt solution (HBSS, Flow Laboratories, Rickmansworth) and the cells resuspended in RPMI 1640 (Flow Laboratories) with 10% fetal calf serum and 25 IU penicillin and 25 $\mu g\,ml^{-1}$ streptomycin. Density gradient centrifugation was used to separate malignant cells from blood stained samples. Two methods were used to disaggregate biopsy samples; mechanical separation by teasing and fine needle aspiration or if necessary the addition of collagenase as described by Bertoncello *et al.* (1982). Once a cell suspension was obtained these samples were also separated by density gradient centrifugation and washed in HBSS before resuspending in culture medium as described above. Cell counts were performed using a haemocytometer and the concentration adjusted to $1 \times 10^6\,ml^{-1}$. The morphology of the cells was assessed prior to plating on a cytospin preparation. The number of malignant cells was also determined by immunocytochemistry using a standard APAAP technique with the monoclonal antibodies HMFG2 (Oxoid, Basingstoke) and Cam 5.2 (Becton-Dickinson, Cowley, Oxford). The viability of the cells was determined by trypan blue dye exclusion.

The relationship of cell numbers to the absorbance of formazan produced was determined by incubation of a range of known cell concentrations with MTT for 4 h. The optimum concentration of MTT and the incubation period to allow adequate formazan production was also determined.

### Drug exposure

Each sample obtained was incubated with up to six different cytotoxic drugs. Stock solutions were prepared in appropriate solvents to a concentration of 100 $\mu g\,ml^{-1}$ (cisplatin, doxorubicin, chlorambucil, mitoxantrone) and 1 $mg\,ml^{-1}$ (carboplatin, treosulfan) and stored at $-20°C$. The drugs were tested at four concentrations appropriate to the plasma levels achieved *in vivo* (Metcalfe, 1983). These were prepared in medium from stock solutions immediately before use. Cisplatin and chlorambucil were used in the range 0.625-5 $\mu g\,ml^{-1}$; doxorubicin and mitoxantrone 0.1-1 $\mu g\,ml^{-1}$: treosulfan and carboplatin 3.12–50 $\mu g\,ml^{-1}$. 100 $\mu l$ aliquots of double strength drug dilution were added to individual wells of a 96 well flat bottom microtitre plate in quadruplicate. 100 $\mu l$ of cell suspension ($1 \times 10^5$ cells per well) were added to the drugs. Control wells containing cells and medium were interspersed throughout the plate. Wells containing medium only were used to blank the spectrophotometer. The cells were continuously exposed to the drugs during a 48 h incubation period in an humidified chamber with 5% $CO_2$/95% air at 37°C.

*MTT assay*

Following incubation the plates were inverted, flicking off the medium and remaining drugs as previously described (Sargent & Taylor, 1989). Fifty $\mu l$ of a 2 $mg\,ml^{-1}$ solution of MTT (Sigma, Poole) in HBSS without phenol red were added to each well and the plates incubated for a further 4 h. Following this the plates were centrifuged for 5 min at 200 **g** and reinverted to remove unconverted MTT, leaving the formazan crystals at the bottom of the well. These crystals were dissolved in 100 $\mu l$ of dimethyl sulphoxide (DMSO) by agitating on a plate shaker for 10 min. The absorbance of the wells was measured using a Dynatech plate reader (MR 600) at wavelength 570 nm. The effect of each drug was determined by calculating the absorbance of the test wells as a percentage of the control wells.

### Results

*Behaviour of cells from ovarian adenocarcinoma in short term culture*

The prepared cell suspensions contained 83 ± 9% malignant cells as determined by morphological assessment of all the cytospin preparations. Using immunocytochemistry, the number of malignant cells in ten (13%) of the cell suspensions was 73 ± 15%. The immunocytochemistry confirmed the morphological assessment and therefore the simpler technique was routinely used. The viability of the tested samples was >75%; this was maintained throughout the test period as assessed by trypan blue dye exclusion and formazan production before and following incubation. Total cell number and percentage of malignant cells in the control samples did not change significantly during the 48 h test period. Mitoses were seen in cytospin preparations of controls both on days 0 and 2. Drug exposure for 2, 4 and 6 days was tested in three patients. However, as found previously with leukaemia cells (Sargent & Taylor, 1989) the viability of control cells was greatly reduced by day 4. An assay duration of 48 h was therefore chosen.

**Figure 1** The relationship between cells/well and the optical density of the formazan produced in two patients (● $r^2 = 0.979$; ○ $r^2 = 0.94$).

**Figure 2** The optical density of formazan produced by $1 \times 10^5$ cells with increasing concentrations of MTT at 1 h (△--△), 2 h (■·····■), 3 h (○---○) and 4 h (●—●).

**Figure 3** The absorbence spectrum of formazan dissolved in DMSO (●—●), 1:3 HBSS in DMSO (□--□) and 1:2 HBSS in DMSO (▲---▲).

*Cell numbers, MTT concentration and duration of incubation*

Figure 1 shows the relationship of cell numbers with formazan production in two patients. It is linear up to $3 \times 10^5$ cells. The median OD of control cells at day 2 plated at $1 \times 10^5$ cells per well was 0.374, range 0.107–1.51. There was no correlation between the OD (i.e. the metabolic activity) of the cells and the number of drugs to which they were sensitive. Incubating $1 \times 10^5$ cells with 100 µg MTT for 4 h gave optimum formazan production (Figure 2).

*Solvent*

Formazan crystals are soluble in acid alcohol (0.04 N HCl in isopropanol) and DMSO. We have previously described the use of acid alcohol to dissolve formazan crystals produced by acute myeloid leukaemic cells (Sargent & Taylor, 1989), but this method is not satisfactory in the assessment of ovarian adenocarcinoma cells. These crystals could only be dissolved in acid alcohol by persistent pipetting which required considerable time and effort. They dissolved easily in DMSO, but as sodium bicarbonate interferes with the optical density of formazan in this solvent (Twentyman & Luscombe, 1987) the unconverted MTT in HBSS must be removed. The plates were therefore centrifuged and re-inverted prior to the addition of DMSO. The alteration of the absorbence spectrum of formazan in DMSO by HBSS is shown in Figure 3. The absorbence peak of the dissolved formazan is 570 nm.

*Biopsy disaggregation*

Collagenase was used in five samples and mechanical disruption in 26. Cell suspensions were achieved in 29; two could not be disaggregated. The two methods produced very different cell viability. Only two samples separated mechanically had a viability of <70%, however all those treated enzymatically had a viability <10%.

*Assessment of cytotoxic drug effect*

Chemosensitivity results were obtained from 51 of the 56 patients (91%). Samples from the remaining five patients were unsuitable for testing: one was contaminated by micro-organisms (biopsy), two did not contain sufficient malignant cells (ascitic fluid) and two did not have satisfactory viability (<70%, biopsy). Overall results were obtained from 63 of 75 samples received, 95% of the ascitic fluid and 75% of the biopsies.

The effects of drugs varied considerably between patients (Figure 4). Weisenthal's criteria (total cell survival, TCS<30% = sensitivity, Weisenthal *et al.*, 1986) for predicting sensitivity *in vivo* in haematological malignancy was applied to these samples. Using this, Table I shows the number of samples sensitive for each drug. A number of samples were sensitive to the platinum and anthraquinone derivatives. Minimal cell kill occurred after exposure to chlorambucil, only one sample showing any degree of sensitivity. Resistance to all the drugs occurred despite no previous administration of the drugs *in vivo*. Eighty-five per cent of the patients were sensitive to at least one drug, the remaining 15% showing resistance to all drugs tested.

Carboplatin and cisplatin produced a similar cell kill in 31 (67%) of 46 samples but the dose of the former was 10 times greater (Figure 5); an observation consistent with other authors when assessing cell line chemosensitivity by clonogenic assay (Hill 1987). Of 26 samples which were resistant to cisplatin, five showed sensitivity to carboplatin and of 30 samples resistant to carboplatin, 10 showed sensitivity to cisplatin.

The effect of doxorubicin was very similar to mitoxantrone in the equivalent dose range in 77% of samples tested against both. Of 24 samples which were resistant to doxorubicin, one was sensitive to mitoxantrone and of 29 resistant to mitoxantrone six were sensitive to doxorubicin.

**Table I** Comparison of cell survival after drug exposure

| *Drug* | *µg ml$^{-1}$* | *Number of patients (%)* TCS<30% (Sensitive) | TCS>30% (Resistant) | *Total* |
|---|---|---|---|---|
| Cisplatin | 5 | 26 (43%) | 34 | 60 |
| Carboplatin | 50 | 16 (35%) | 30 | 46 |
| Doxorubicin | 1 | 17 (31%) | 37 | 54 |
| Mitoxantrone | 1 | 6 (17%) | 30 | 36 |
| Treosulfan | 50 | 8 (23%) | 27 | 35 |
| Chlorambucil | 5 | 1 ( 2%) | 48 | 49 |

TCS, total cell survival.

**Table II** Reproducibility of the MTT assay in four patients

| *Patient* | *Drug concentration (µg ml$^{-1}$)* | | *TCS % (s.e.m.)* Assay 1 | Assay 2 |
|---|---|---|---|---|
| DC | Cis | 0.625 | 69(13) | 73(8) |
| | | 1.25 | 57(8) | 50(8) |
| PT | Cis | 0.625 | 97(10) | 90(10) |
| | | 1.25 | 90(4) | 90(6) |
| | Dox | 0.5 | 39(3) | 35(4) |
| | | 0.75 | 18(3) | 14(4) |
| | Chl | 0.625 | 93(8) | 97(2) |
| | | 1.25 | 105(5) | 108(5) |
| AG | Cis | 0.625 | 100(10) | 76(10) |
| | | 1.25 | 84(3) | 86(12) |
| PA | Cis | 0.625 | 80(2) | 94(7) |
| | | 1.25 | 89(7) | 87(3) |
| | Dox | 0.5 | 16(2) | 28(8) |
| | | 0.75 | 12(5) | 12(5) |
| | Chl | 0.625 | 110(18) | 89(3) |
| | | 1.25 | 90(9) | 88(13) |

Cis, cisplatin; Dox, doxorubicin; Chl, chlorambucil; TCS, total cell survival.

The reproducibility of the assay is shown in Table II. In patients DC and PT the assay was performed on two occasions using the same sample. Two separate samples were obtained from patients AG and PA; the first at diagnostic paracentesis and the second at ensuing laparotomy.

### Discussion

The aim of these experiments was to assess the feasibility of using the MTT assay for chemosensitivity testing of individual patients with ovarian malignancy. We have shown that the criteria necessary for validation of the MTT assay can be fulfilled using cells derived from malignant effusions and biopsy samples from patients with ovarian cancer. Single cell suspensions with good viability suitable for analysis could be obtained and required little preparation before drug incubation. The relatively small number of cells required, $1-2 \times 10^6$ for each drug, allows assessment of a range of concentrations of many cytotoxic agents. A good, reproducible variation in drug effect between patients is obtained after only 48 h incubation. Each patient had an individual drug resistance and sensitivity pattern. This drug sensitivity profile could offer the clinician the opportunity to select an active agent in individual cases. Testing of primary tumour samples has this obvious advantage but could, however, present some technical difficulties. Contamination by metabolically active non-malignant cells capable of reducing MTT to formazan could falsify results. In our study one sample was contaminated by micro-organisms but this was easily identified before addition of MTT as all plates are routinely examined by inversion microscopy before each step of the assay. Although non-malignant cells were present, all samples had a high percen-

**Figure 4** Log dose–response curves for six drugs showing variation in sensitivity between patients. Cisplatin, $n = 17$; carboplatin, $n = 16$; doxorubicin, $n = 14$; mitoxantrone, $n = 11$; treosulfan, $n = 6$; chlorambucil, $n = 16$.

**Figure 5** Comparison between drug analogues. Log dose–response curves in two patients for: (**a**) doxorubicin (solid lines) and mitoxantrone (dotted lines); (**b**) cisplatin (solid lines) and carboplatin (dotted lines).

tage of malignant cells. The biopsy samples were of poorly differentiated tumours and consequently contained a high ratio of malignant/stromal cells. Lymphocytes were the main non-malignant contaminant. Their numbers were small and like other authors we found that their metabolic activity was low (Pieters *et al.*, 1988; Hongo *et al.*, 1987). Occasional fibroblasts were seen but in a 48 h culture, plate overgrowth did not occur. Our friable tumour samples lent themselves to mechanical disaggregation thus obviating the need for enzymatic disaggregation, a method we found disappointing despite previous favourable reports (Courtenay, 1983). Ovarian cancer cells tend to cluster together in the microtitre plate wells. These small groups of cells create difficulty in assays which require direct cell counting. They may also be misinterpreted as colonies in clonogenic assays (Bertoncello *et al.*, 1982). By measuring cellular metabolic function in the MTT assay these problems are circumvented.

The data presented suggest that drug selection could be aided in 91% of patients with ovarian cancer. The platinum derivatives, currently the first line treatment in the management of ovarian malignancy, produced the greatest cytotoxic effect in our test system. The 43% response rate found to cisplatin is closer to the complete clinical response rate reported by Wiltshaw *et al.* (1986) than that of 16% reported by Welander *et al.* (1983) using the clonogenic assay. The cross-resistance pattern between the effects of cisplatin and carboplatin also agrees with that reported from clinical observations (Wiltshaw, 1985). The sensitivity rate to chlorambucil, however, is lower than the expected value for complete clinical remission (24% Australian Gynaecological Oncology Group, 1986; 15% Williams *et al.*, 1985). This may be due to the *in vitro* concentration of chlorambucil being too low. We chose this range because of peak plasma levels but perhaps a higher concentration range which shows maximum variation between patients as proposed by Bosanquet's group (Bird *et al.*, 1988) would be more appropriate in this assay.

As surgery is the mainstay of treatment in early ovarian cancer (87% 5 year survival FIGO stage 1a, Dembo *et al.*, 1979), it is in advanced disease that drug screening offers the greatest benefits. It is of interest that the decision to commence chemotherapeutic agents in recurrent early disease may depend on the presence of malignant cells in peritoneal washings obtained at second look laparoscopy or laparotomy (Wiltshaw, 1981). Such samples would be ideally suited to this assay.

The true value of the MTT assay in ovarian malignancy will only be apparent if the *in vitro* results predict *in vivo* response. In view of the variation in drug effect between patients, the proximity to clinical response rates, the ease of testing and high percentage of successful assays, we believe this method is suitable for such clinical trials.

### References

ALBERTS, D.S., CHEN, H.S.G., SOEHNLEN, B. & 4 others (1980). In-vitro clonogenic assay for predicting response of ovarian cancer to chemotherapy. *Lancet*, **ii**, 340.

ALLEY, M.C. & LIEBER, M.M. (1984). Improved optical detection of colony enlargement and drug cytotoxicity in primary soft agar cultures of human solid tumour cells. *Br. J. Cancer*, **49**, 225.

ALLEY, M.C., SCUDIERO, D.A., MONKS, A. & 7 others (1988). Feasibility of drug screening with panels of human tumour cell lines using a microculture tetrazolium assay. *Cancer Res.*, **48**, 589.

AUSTRALIAN GYNAECOLOGICAL ONCOLOGY GROUP (1986). Chemotherapy of advanced ovarian adenocarcinoma: a randomised comparison of combination versus sequential therapy using chlorambucil and cisplatin. *Gynecol. Oncol.*, **23**, 1.

BERTONCELLO, I., BRADLEY, T.R., CAMPBELL, J.J. & 6 others (1982). Limitations of the clonal agar assay for the assessment of primary human ovarian tumour biopsies. *Br. J. Cancer*, **45**, 803.

BIRD, M.C., BOSANQUET, A.G., FORSKITT, S.& GILBY, E.D. (1988). Long-term comparison of results of a drug sensitivity assay in vitro with patient response in lymphatic neoplasms. *Cancer*, **61**, 1104.

BLACK, M.M. & SPEER, F.D. (1954). Further observations on the effects of cancer chemotherapeutic agents on the in vitro dehydrogenase activity of cancer tissue. *J. Natl Cancer Inst.*, **14**, 1147.

CARMICHAEL, J., DE GRAFF, W.G., GAZDAR, A.F., MINNA, J.D. & MITCHELL, J.B. (1987). Evaluation of a tetrazolium-based semiautomatic colorimetric assay. *Cancer Res.*, **47**, 936.

CARNEY, D.N. & WINKLER, C.F. (1985). In vitro assays of chemotherapeutic sensitivity. In *Important Advances in Oncology*, Devita *et al.* (eds) p. 78. J.B. Lippincott: Philadelphia.

COURTENAY, D. (1983). The Courtenay clonogenic assay. In *Human Tumour Drug Sensitivity Testing in Vitro*, Dendy, P.P. & Hill, B.T. (eds) p. 103. Academic Press: London.

DEMBO, A.J., BUSH, R.S., BEALE, F.A., BEAN, H.A., PRINGLE, J.F. & STURGEON, J.F.G. (1979). The Princess Margaret Hospital study of ovarian cancer: stage I, II and asymptomatic stage III presentations. *Cancer Treat. Rep.*, **63**, 249.

HILL, B.T. (1983). An overview of correlations between laboratory tests and clinical response. In *Human Tumour Drug Sensitivity Testing in Vitro*, Dendy, P.P. & Hill, B.T. (eds) p. 235. Academic Press: London.

HILL, B.T. (1987). In vitro screening of new drugs and analogues – specificity and selectivity. *Cancer Treat. Rev.*, **14**, 197.

HONGO, T., FUJII, Y., MIZUNO, Y., HARAGUCHI, S. & YOSHIDA, T.O. (1987). Anticancer drug sensitivity test using the short-term microplate culture and MTT dye reduction assay. *Jpn. J. Cancer Chemother.*, **14**, 472.

METCALFE, S.A. (1983). A review of methods for estimating clinically achievable antitumour drug levels and their association with studies in vitro. In *Human Tumour Drug Sensitivity Testing in Vitro*, Dendy, P.P. & Hill, B.T. (eds) p. 213. Academic Press: London.

MOSMANN, T. (1983). Rapid colorimetric assay for cellular growth and survival: application to proliferation and cytotoxic assays. *J. Immunol. Methods*, **65**, 55.

PIETERS, R., HUISMANS, D.R., LEYVA, A. & VEERMAN, A.J.P. (1988). Adaption of the rapid automated tetrazolium dye based (MTT) assay for chemosensitivity testing in childhood leukaemia. *Cancer Lett.*, **41**, 323.

SARGENT, J.M. & TAYLOR, C.G. (1989). Appraisal of the MTT assay as a rapid test of chemosensitivity in acute myeloid leukaemia. *Br. J. Cancer*, **60**, 206.

TWENTYMAN, P.R., FOX, N.E. & REES, J.K.H. (1989). Chemosensitivity testing of fresh leukaemic cells using the MTT colorimetric assay. *Br. J. Haematol.*, **71**, 19.

TWENTYMAN, P.R. & LUSCOMBE, M. (1987). A study of some variables in a tetrazolium dye (MTT) based assay for cell growth and chemosensitivity. *Br. J. Cancer*, **56**, 279.

WELANDER, C.E., HOMESLEY, H.D. & JOBSON, V.W. (1983). In vitro chemotherapy testing of gynecologic tumors: basis for planning therapy? *Am. J. Obstet. Gynecol.*, **147**, 188.

WEISENTHAL, L.M. & LIPPMAN, M.E. (1985). Clonogenic and nonclonogenic chemosensitivity assays. *Cancer Treat. Rep.*, **69**, 615.

WEISENTHAL, L.M., DILL, P.L., FINKLESTEIN, J.Z., DUARTE, T.E., BAKER, J.A. & MORAN, E.M. (1986). Laboratory detection of primary and acquired drug resistance in human lymphatic neoplasms. *Cancer Treat. Rep.*, **70**, 1283.

WILLIAMS, C.J., MEAD, G.M., MACBETH, F.R. & 8 others (1985). Cisplatin combination therapy versus chlorambucil in advanced ovarian carcinoma: mature results of a randomized trial. *J. Clin. Oncol.*, **3**, 1455.

WILTSHAW, E. (1981). The management of stage I carcinoma of the ovary. In *Progress in Obstetrics and Gynaecology, Volume I*, Studd, J. (ed.) p. 229. Churchill Livingstone: Edinburgh.

WILTSHAW, E. (1985). Ovarian trials at the Royal Marsden. *Cancer Treat. Rev.*, **12**, 67.

WILTSHAW, E., EVANS, B., RUSTIN, G., GILBEY, E., BAKER, J. & BARKER, G. (1986). A prospective randomized trial comparing high-dose cisplatin with low-dose cisplatin and chlorambucil in advanced ovarian carcinoma. *J. Clin. Oncol.*, **4**, 722.